# Mercury vapor volatilization from particulate generated from dental amalgam removal with a high-speed dental drill – a significant source of exposure

**DOI:** 10.1186/s12995-019-0240-2

**Published:** 2019-07-17

**Authors:** David Warwick, Matt Young, Joe Palmer, Robin Warwick Ermel

**Affiliations:** 1 Dental Practice, Hanna Dental Clinic, 202, 2nd Ave W, Hanna, Alberta Canada; 2 Dental Practice, 728 5th Ave. West, Hendersonville, NC USA; 3 Dental Practice, Palmer Distinctive Dentistry, 134 Milestone Way, Greenville, SC USA

**Keywords:** Mercury, Mercury vapor, Dental amalgam, Occupational exposure to mercury, Amalgam removal

## Abstract

**Background:**

The ubiquitous use of dental amalgam for over 180 years has resulted in the exposure of millions of dental workers to mercury. Dental amalgam contains approximately 50% mercury. Dental workers, including dentists, dental assistants, and dental hygienists, have been shown to have increased levels of mercury and suffer more from health issues related to mercury exposure than the general public. Mercury is known to be absorbed via inhalation or through the skin. There are many routine dental procedures that require the removal of dental amalgam by using the dental high-speed drill, which we suspected generates an occupational mercury exposure that is not sufficiently recognized.

**Results:**

We showed that drilling dental amalgam generates particulate that volatilizes significant amounts of mercury vapor generally for more than an hour after removal. The levels of mercury vapor created by this procedure frequently exceed the safety thresholds of several jurisdictions and agencies.

**Conclusions:**

A significant, underrecognized source of localized exposure to mercury vapor was identified in this study. The vapor was created by microgram levels of particulate generated from dental amalgam removal with a high-speed dental drill, even when all feasible engineering controls were used to reduce mercury exposure. This exposure may explain why dental workers incur health effects when safety thresholds are not breached. The dispersion patterns for the particulate are not known, so the use of effective skin barriers and inhalation protection are required during amalgam removal to protect the dental worker from this form of occupational mercury exposure. Standard methodologies for occupational mercury exposure assessment appear to be inadequate when assessing mercury exposure during amalgam removal.

**Electronic supplementary material:**

The online version of this article (10.1186/s12995-019-0240-2) contains supplementary material, which is available to authorized users.

## Background

We hypothesized that the action of drilling amalgam with a dental high-speed hand piece, even when using protocols and all feasible engineering controls to minimize mercury vapor, would still generate an aerosol of particles that would be heated sufficiently to produce increased mercury vapor. We designed the study to answer the following questions:

- What concentration of mercury vapor can be reached from particulate generated from the removal of dental amalgam restorations using a high-speed drill?
- How long can the particulate volatilize mercury vapor?
- Is the peak vapor generated associated with the mass of the mercury in the particulate?
- Does the amount of amalgam removed in each sample affect the peak Hg vapor?
- Does the amount of amalgam removed in each sample affect the mass of mercury in particulate collected?

Amalgam fillings are a widely used dental restorative material and have been utilized since the nineteenth century. Use of the material is declining in the developed nations but is increasing in the developing countries. It has been estimated that global use of mercury for dental amalgam in 2015 was 226–322 tones [1]. The longevity of an amalgam filling has a very large range but is expected to provide service on average for 10 years. After the amalgam’s service period has ended, removal is required which is generally achieved using a high-speed drill. There are other indications where dental amalgam removal is required and these are listed later in the background. Dental amalgam consists of approximately 50% mercury and 50% base metals. The toxicity of mercury is well established.

Dental workers, including dentists, dental assistants, dental hygienists, dental students, dental instructional staff, and dental laboratory and sterilization technicians are all at risk of mercury exposure if they work with dental amalgam [2–15]. Dental associations and dental schools have specific policies on the use of amalgam because of its mercury content. Additionally, Safety Data Sheets or SDS (formerly Material Safety Data Sheets or MSDS) from manufacturers of amalgam outline the risk of mercury exposure when using amalgam.

Dental workers have higher levels of mercury [3, 5, 10, 12, 16–18] as measured in blood, urine, stool, nails, hair and organs. Dental workers also have a higher prevalence of health issues consistent with chronic mercury exposure than controls. These health problems include adverse neurological conditions [2, 3, 6–9, 17, 19], while there are also some possible indications that exposure to elemental mercury may also affect reproduction [20–22].

Mercury levels either in the tissue of the dental worker or in the dental working environment have been found to be lower than established safety levels. It has, however, also been argued that adverse health effects can occur at such levels and after many years [23]. In a Swedish study conducted to assess mercury exposure and health effects in dental personnel, the researchers found that the mean mercury levels in the personal space of 44 dental workers were well below established safety thresholds. The urinary mercury levels of this group were not elevated when compared to a control group. Despite the unremarkable levels of mercury measured in air and urine, central nervous symptoms of the dental group were significantly higher than the controls [24]. This suggests that the current safety limits may be too lenient.

Although many jurisdictions have set biological exposure indexes, it was concluded within a review from 2012 that “it has not been possible to set a level for mercury in blood or urine below which mercury related symptoms will not occur [25].” Others claim that there is an unreliability in the current methods to measure various tissue samples to determine mercury exposure [17]. Further, WHO has stated: “Recent studies suggest that mercury may have no threshold below which some adverse effects do not occur.” [26]

It is certain that there are susceptible subsets of the population that are more likely to be affected by chronic mercury exposure. In a recent study of the effects of mercury in a cohort of children, genetic polymorphisms were identified that made the participants more susceptible to mercury [27], and additional research has explored this pertinent genetic component [28]. Specifically, the role of genetic profiles in dental workers’ reactions to mercury has been examined [7–9].

The World Dental Federation (FDI) recommends avoiding direct skin contact with mercury or freshly mixed dental amalgam and avoidance of mercury vapour sources including during the removal of dental amalgam [29]

It has been claimed that the respirable particulate matter represents the largest share of daily Hg-exposure for the practicing dentist [14]. By use of standard exposure assessment methods it was found that a dentist who removes four amalgam fillings per day will inhale 38 mg of mercury derived from amalgam particulate, by far exceeding any level considered safe. When respirable amalgam particles are deposited in the lungs, they reach body temperature that enhances vaporization over days and thus, also subsequent absorption.

It is important to understand that although particulate is the exposure source, it is the vapor that comes off the particulate that is of interest because in this form, it is very easily taken into the body by the lungs and the skin. The amount of Hg vapor from amalgam increases with stimulus [15, 30, 31]. These stimuli generally cause an increase in temperature which increases the vapor pressure. The dental high-speed drill can spin up to speeds of 350,000 rpm [32], and therefore, can generate friction and increase the heat of the material being removed.

As long as installation of amalgam continues (and for years after it ends), there will be a need to remove amalgam from teeth. There are several circumstances that require the removal of dental amalgam from teeth using a high-speed dental drill. These include, but are not limited to, the following scenarios: sectioning of a tooth to facilitate dental extraction, failed seal of an existing amalgam restoration, recurrent decay under a filling, fracture of a tooth with an amalgam filling, adjustment of an incorrect bite, preparation for a fixed or removable prosthesis, root canal access opening, reshaping of an existing amalgam, removal of an amalgam that has an open inter-proximal contact, removal to prevent galvanism with another intra-oral metal, removal for health reasons, removal to reduce exposure of mercury, treatment of periodontal disease, and removal due to mercury sensitivities.

The guidelines for assessing mercury presence in the work place is outlined by OSHA under Method Numbers ID-140 [33] for vapor and ID-145 [34] for particulate. The complete processes suggested in these documents are complicated and beyond the scope of this paper; however, there are some specific items in the methods that are pertinent to this study. The assessment of mercury in the workplace is accomplished by using three different techniques. The first is the use of a passive or active mercury vapor sampling device for atmospheric mercury vapor levels. The second is the use of a vacuum with a filter cassette to collect mercury containing particulate in the air, and the third method is the use of wipes to collect mercury containing particulate on surfaces.

There are several drawbacks to these methods with respect to assessing mercury exposure from amalgam particulate generated by drilling. Vapor samplers do not assess particulate or localized mercury vapor generated from particulate if the sensors are in a location that does not have access to the particulate. If particulate in air is assessed using the vacuum filter cassettes, according to procedure described in OSHA ID-145 section 5.4, the cassettes are to be sealed after taking the sample and sent to the lab to determine the mass of the mercury in the particulate. The cassette does not allow access to the filter in a way that assessment of mercury vapor from this particulate can be made. The standardized wiping technique for determining particulate on surfaces as outlined by OSHA ID-145 states that a wet gauze is used to wipe a 10 cm × 10 cm square. The most concerning surfaces that require assessing for particulate presence are the potential skin areas that may be exposed to the amalgam particulate. This is particularly true because skin is a known route of absorption for mercury. The shape of the hands, arms, face, chest and other parts of the dental worker’s and dental patient’s anatomy that may be exposed to amalgam particulate during dental operations do not lend themselves to classical surface wiping. When considering all of these drawbacks with respect to standard occupational assessment protocol for mercury exposure in dentistry, it is evident that these processes do not quantify the extent of mercury exposure.

There is very little information on the levels of mercury vapor that can be emitted from fresh amalgam particulate generated from the dental high-speed drill. There are two limitations that may have prevented this effort. The first is that the respirable particulate is inhaled and not available for measurement of vapor because it is inaccessible to measuring devices. Second, as mentioned previously, current OSHA standards to collect particulate actually prevent the volatilization measurements because the concern of particulate assessment is to determine the mass of the mercury in the particulate, not the vapor emitting from the particulate.

There are others who have aimed to measure exposure by amalgam removal, but have failed to quantify mercury vapour from the particle matter generated [15, 35–38].

While these and other studies have examined the role of the dental drill in generating mercury releases [15, 35–40], it appears as though there have been no detailed attempts in the scientific literature to quantify the level of mercury that may vaporize from the surface of freshly ground dental amalgam particulate. By doing so, perhaps an under-estimated occupational mercury exposure in dentistry can be identified. This is the aim of this study.

Mercury vapor can be absorbed via inhalation and skin [41]. It is these two routes of absorption of mercury that prompted the design of this study. Assessing the concentration of localized mercury vapor that the skin or the lungs would endure after particulate exposure required measuring the vapor derived from particulate at as close a range as reasonably possible. In this methodology, we aim to illustrate the potential mercury vapour exposure from particulate that comes into contact with these two organ systems.

## Method

There were no ethical implications of the methodology of this study as described in the “Declarations” section of this paper.

Amalgam particulate was collected in-vivo from 21 patients who were scheduled for amalgam removal in the dental offices of Dr. David Warwick and Dr. Matthew Young during a time period starting October 2016 and April 2018. The particulate was sampled from the head of the dental drill with a 5 cm × 5 cm (2 × 2) cotton wipe during a regularly scheduled clinical procedure. No procedure was performed on any patient that deviated from the standard of care. The following controls were in place to minimize drilling, particulate formation, and mercury vapor generation and exposure:

- copious amounts of water
- reduced drilling of the amalgam by cross hatching the material and removing bulk pieces.
- high volume suction with custom isolation tip (Clean Up brand)
- secondary air evacuation (additional venting to the dental suction providing approximately 9–20 cubic meters per minute laminar air flow evacuation through a 8–12 cm hose from the operative site exhausted to the outside or through a set of mercury rated filters) eg. Dentair Vac.
- non-latex dental dam on the patient
- full facial and body barrier for patient
- patient saliva suction behind the rubber dam
- alternative air supply to patient.
- face shield, mercury rated gown and head protection, nitrile gloves, and mercury rated breathing protection for dentist and assistant

Immediately after the removal of the dental amalgam/s from patients’ mouths, a sample of particulate was obtained by wiping the head of the dental drill with a 2 × 2 gauze. The head of the drill is a predictable area where particulate accumulates which makes it a convenient place to collect from. The 2 × 2 gauze containing the particulate was then placed 1 cm from the inlet hose of a recently calibrated Mercury Instruments Mercury Vapor Monitor 3000 (Model VM 3000) and 1 s increment readings of the localized mercury vapor concentration were recorded. The VM 3000 evaluates incoming gas for the presence of Hg using Atomic Absorption Spectroscopy (AAS) and is considered an industry standard in the field of mercury monitoring.

The authors also made note of the relative size of the amalgams being removed as large, medium, and small, and the number of amalgams removed in the session to generate a “Total Filling Size Score”. The mercury vapor measurements were generally taken until the localized level fell below 10 μg/m3, although there were samples that did not drop to this level even after several hours of measurement.

After the vapor measurements were taken, the wipe containing the particulate was placed in a container using new gloves and shipped to AGAT labs in Canada. AGAT labs are an analytical lab certified to ISO 9001, ISO/IEC 17025 standards and is accredited with the following organizations; SCC, CALA and QMI-SAI Global. The mass of the mercury in each sample sent to AGAT was determined using ICP/MS analytical technique.

Two dentists (Warwick and Young) provided each 1 control and 16 and 5 patient samples respectively.

For each patient sample, we generated the following data:

1. the approximate size and number of the fillings that were removed depicted as a “Total Filling Size Score”
2. one second incremental readings by the VM 3000 which measured localized Hg vapor levels in μg/m3 vaporizing from the amalgam particulate
3. peak Hg vapor
4. the mass of Hg contained in each sample

Additionally, one of the dentists provided measurements of the mercury vapor that evaporated from 400 mg of room temperature (20 °C) elemental mercury (the mass of the elemental mercury was not measured but derived from the manufacturers data sheet on the Dispersalloy product) with an approximate surface area of 0.07 cm^2^ from a Dispersalloy single spill dental amalgam capsule over a 33-min time period to compare with the readings from the particulate. This size of the elemental sample was used as it was readily available from a conventional unmixed amalgam capsule.

Only one elemental mercury sample was taken for this study as the results from this measurement fell within expected theoretical levels of mercury evaporation. In our sample, the average concentration for the first ½ hour of the vapor expressing from the elemental mercury was 35 μg/m^3^. The VM 3000 has a flow rate of approximately 80 l/hour or 0.08 m^3^/h. The mass of mercury that evaporated from the elemental mercury was 35 μg/m^3^ x ½ × 0.08 m^3^/h or 1.4 μg/h. The evaporation rate of elemental mercury at room temperature (20 C) is approximately 50 μg/cm^2^/h (range of 40–60 μg/cm^2^/h) [42]. In the theoretical model, we would expect that the elemental mercury we measured, which had an estimated surface area of 0.07 cm2 (radius of .15 cm), would evaporate .07 cm2 x ½ hour × 50 μg/cm^2^/h or 1.75 μg/h. It is most common to see experimental measures of mercury evaporation from elemental mercury to be lower than theoretical proposals. The most likely cause of this is oxidation of the surface of the mercury, which reduces the evaporation rate. The phenomenon of a decreasing volatilization of mercury from our sample over time is likely due to oxidation as well.

## Results

None of the two control samples generated Hg vapor as monitored by the VM 3000 and had less than detectable μg of Hg mass as measured by AGAT labs. Each filling removed in the process was rated by the dentists (Warwick and Young) as small, medium or large and was given a score of 1, 2 or 3 respectively. A “Total Filling Size Score” for each session was then determined by summing the score of each filling removed in the session (Table 1).

**Table 1:**
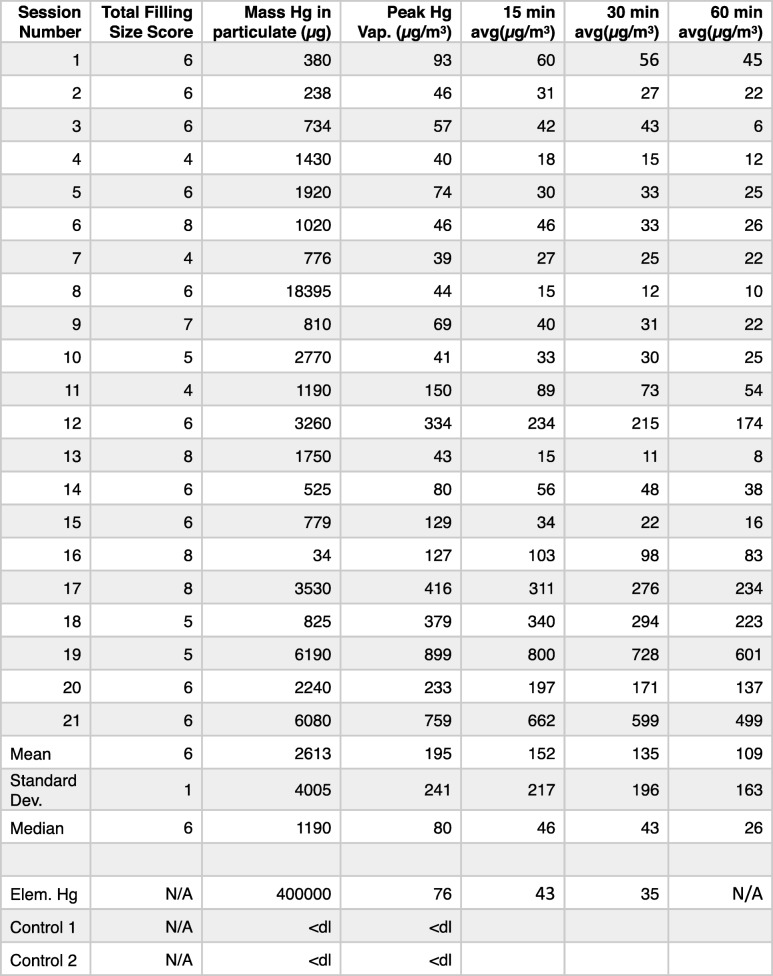
Total Filling Size Score, volatilization readings and mass of mercury containing particulate (*n* = 21) and mass and volatilization readings of controls (*n* = 2) and an elemental mercury sample (*n* = 1)

Details size rating of the fillings that were removed, the peak and 15-min, 30 min and 60-min average Hg vapor readings, and the mass of mercuryin each sample. It also lists the peak and 15-min and 30-min average Hg vaporthat was vaporized by the elemental mercury. The table further indicates the Hg massand vapor results of the control wipes, which were below detectable limits (<dl)

Interestingly, the total mass of all the particulate collected in the 21 cases was 54,876 μg, which represents only about 10% of the mercury in an average sized filling and only about 3–4% of the average mercury content of the amalgams removed in a single session.

## Discussion

In order to understand the impact of the mercury sources identified in this study, an understanding of standardized safety levels or threshold limit values (TLV) is required. Occupational safety levels vary in different jurisdictions and in different administrations. In Table 2, reference levels from the Occupational Safety and Health Administration (OSHA)(U.S.), The National Institute for Occupational Safety and Health (NIOSH)(U.S.), Association Advancing Occupational and Environmental Health/American Conference of Governmental Industrial Hygienists (ACGIH), and Alberta (Canada) Occupational Health and Safety (AOHS) are listed. The Alberta Code is included because the majority of samples were obtained in Alberta, Canada.

**Table 2:**
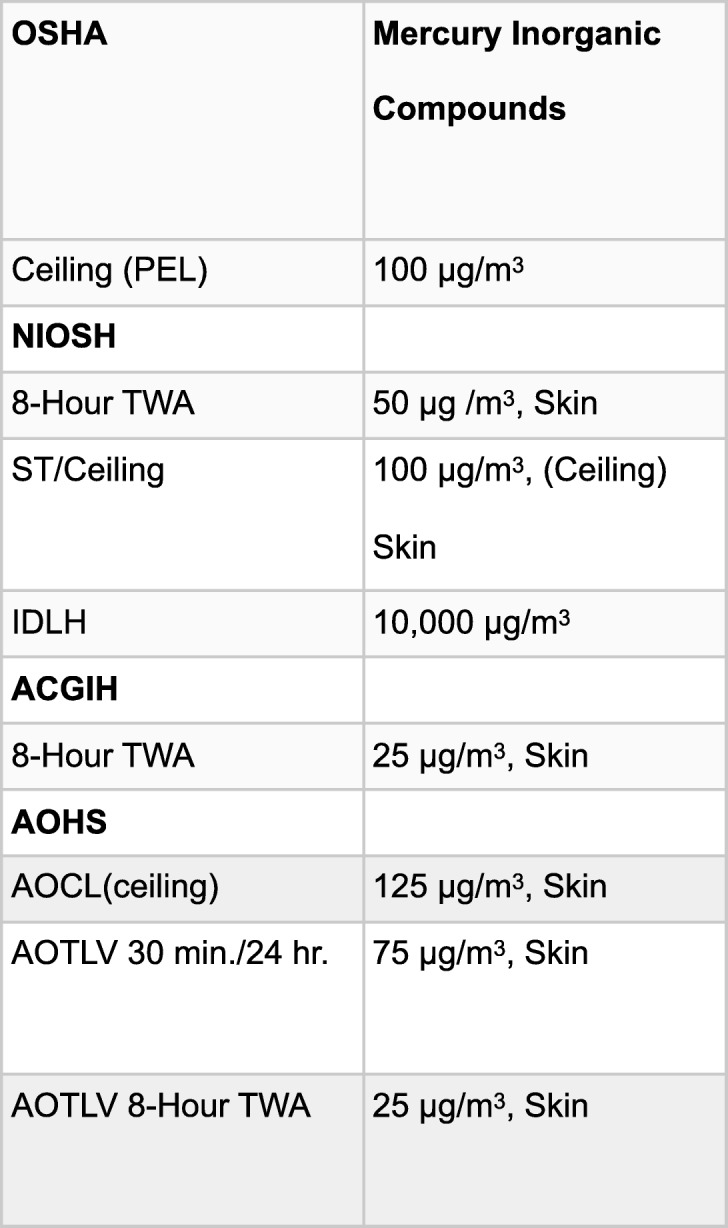
Occupational threshold levels from OSHA, NIOSH, ACGIH, and AOHS

Various occupational threshold levels referenced from the OSHA Website (https://www.osha.gov/SLTC/mercury/standards.html) and GoAB (Government of Alberta) Occupational Health and Safety Act, Occupational Health and Safety Code. Edmonton, AB: Government of Alberta; 2009 illustrate the inconsistency of various responsible agencies.

All of these groups acknowledge the ability of Hg vapor to be absorbed by the lungs and skin. Beyond this consistency, one can identify many differences these bodies have with regard to safety levels of occupational Hg exposure.

**Fig. 1:**
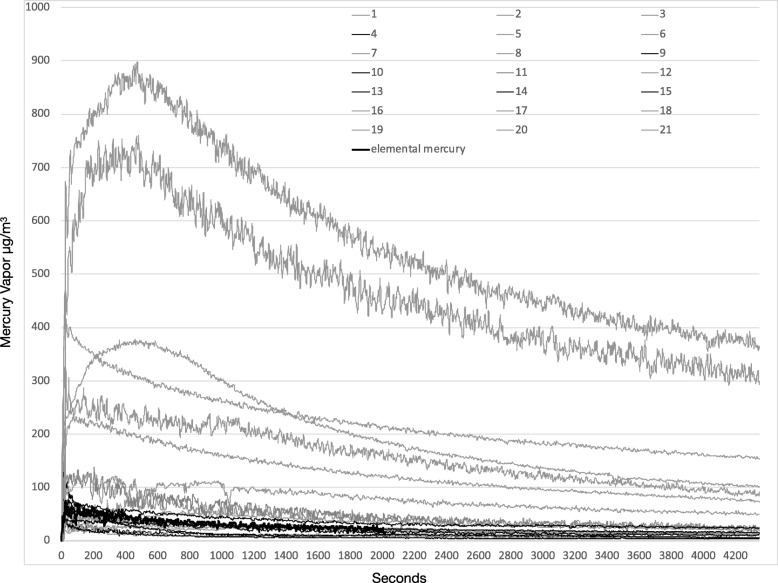
Mercury volatilization rates (μg/m^3^) over time of 21 particulate samples and 1 elemental mercury sample

**Fig. 2:**
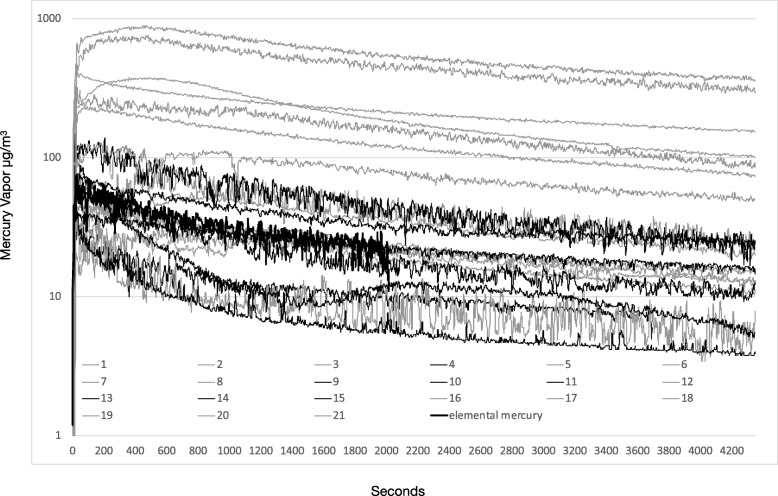
Mercury volatilization rates (μg/m^3^) expressed in a logarithmic axis over time of 21 particulate samples and 1 elemental mercury sample

There are several observations from Figs. 1 and 2 that are pertinent to occupational safety.

### Ceiling Thresholds

The first is the high levels of Hg vapor that are generated from amalgam particulate collected from the head of the drill by wiping with a 2 × 2 gauze. The arithmetic mean of the peaks of the 21 samples (195.1 μg/m^3^) were substantially higher than OSHA’s Permissible Exposure Limit (PEL) level and NIOSH’s ceiling level of 100 μg/m^3^ and AOHS’s ceiling of 125 μg/m^3^. Nine of the 21 peak levels breached both NIOSH’s and AOHS’s ceiling level. The median of the peak of the 21 readings was 80 μg/m^3^, which is under the ceiling levels described in this section.

### Average Thresholds

Seven of the twenty-one 30-min averages breached the AOHS 30 min/24-h threshold, with another sample coming in just under the 75 μg/m^3^. In 4 of the 21 samples taken, the 60-min average was high enough that even if there was no other exposure for another 7 h, the time weighted 8-h average would still breach the 25 μg/m^3^.

**Fig. 3:**
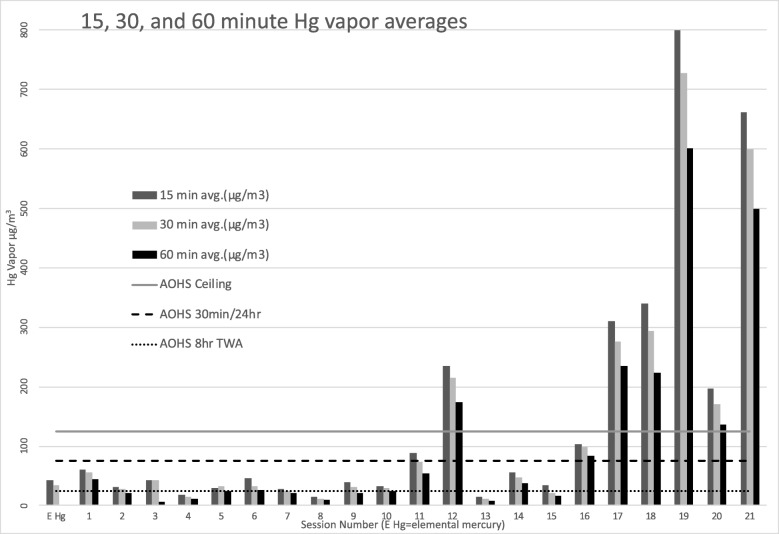
Fifteen, 30- and 60-min Hg vapor averages of the 21 particulate samples and 15 and 30 Hg averages of the elemental mercury sample

In Figs. 1, 2 and 3, we observed an expected reduction of the mercury vapor levels over time due to anticipated cooling off of the particulate. It is also reasonable to assume that the surface may oxidize over time which also can reduce volatilization. It is clear that dental particulate generated from drilling amalgam fillings produces mercury vapor at levels of concern over a substantial period of time that can breach threshold levels and that this needs to be considered in occupational safety strategies.

It is also apparent that the peak and average vapor readings from room temperature elemental mercury were much lower than corresponding particulate readings. This was despite the fact that the mass of elemental mercury used was at least 100 times greater than any of the samples of particulate.

The surface area of mercury, being a factor in the rate of volatilization, is a calculation that we were unable to assess with respect to particulate since the surface area of the particulate is unknown. Because the levels of mercury vapor generated by particulate was up to 1 order of magnitude higher than the levels generated by room temperature elemental mercury at masses 2 orders of magnitude less, particulate must be considered a dental waste that is more toxic than elemental mercury. Skin contact and inhalation must be avoided.

As well, several of the samples peak levels breached the stated ceiling thresholds by more than 7-fold. It must be understood that these thresholds are based on a general exposure, and the mercury sources from particulate that we have identified may not qualify for this protective limit, as these sources represent a localized source of acute mercury exposure.

Another observation from the data is the amount of time that the particulate emits Hg vapor, especially when one considers the very low mass. Although not shown in Figs. 1 or 2, some of the larger mass samples continued to off gas mercury vapor at levels above ceiling thresholds (100–125 μg/m3) for over 4 h. The other implication of the extended time that the samples off-gas mercury vapor is that this exposure may increase the risk of time weighted exposure limits that have been established.

The greatest share of the amalgam particulate generated by high-speed dental drills is in the fully respirable range [14]. There are several models that suggest that the lungs provide an environment where the volatilization of Hg vapor from particulate would be at least as favorable as particulate at room temperature, since the rate of volatilization is dependent on temperature [14]. Unfortunately, once these particles are inhaled, they are hidden by any occupational measuring technique. However, one could recognize the potential for an extended mercury exposure in the lungs that could last at least hours after the inhalation of amalgam particulate. The inhalation of particulate would then decrease the ability of occupational measurements to be made because the volatilization is occurring in the dental worker’s body.

Particulate that lands on the skin can also provide a mercury source that may not be properly considered in occupational assessment. The Hg vapor from particulate is a very localized plume of Hg vapor that can absorb through the skin; however, if the skin as a surface is not measured locally, there may be an understated exposure level if, for example, ambient room air is assessed.

There is limited information regarding mercury absorption via the skin in the literature, and essentially no information specifically on volatilizing dental amalgam particulate.

Hursh et al. of the Environmental Health Sciences Center in Rochester, New York, measured human percutaneous absorption of mercury vapor at concentrations of 0.88–2.14 ng/cm^3^ and estimated that the skin of the forearm will absorb approximately 2.2% that of the lungs at the same concentration of mercury vapor [43]. This testing showed that 216–844 ng of mercury was absorbed over a 27 to 43 min. Half of this was shed by desquamation, and the other half made it into the circulation and could be measured systemically. Given that the conversion of ng/cm^3^ to μg/m^3^ is 1:1000, the vapor generated by amalgam particulate is less than Hursh et al.’s source by a factor of 10. The median of Hursh et al.’s exposure level would be 1.51 ng/cm^3^ or 1510 μg/m^3^, as compared to the median peak levels of 80.1 μg/m^3^ in particulate group.

The particulate that was collected in this study was a small fraction of the potential amount of particulate generated, and a much larger amount has the potential to land on the skin proximal to the operative site such as the hands, wrists, forearms, face, neck and even lap and thighs of the dental worker. Multiple skin destinations of particulate could theoretically increase the potential exposure of mercury via this route. If a dental worker has bare skin or clothing that is permeable to Hg vapor, particulate on the skin or in the non-protective clothing can continue to provide a source of mercury vapor to the skin for hours.

There is a risk of skin absorption of mercury vapor from volatilizing particulate during and well after amalgam removal, and as a result, it would make sense for dental workers to wear skin barriers as the amalgam is being removed. It also makes sense, because of the extended time that the particulate emits mercury vapor, to remove the protective barriers and clothing once the amalgam removal is complete and conscientiously dispose of this contaminated gear outside the confines of the dental office.

It is important to understand that because the collection of samples was collected in vivo, the particulate was generated while using all the techniques known to minimize Hg vapor production. Warwick [15], Nimmo [35], and Brune [37] all confirmed a dramatic decrease in mercury vapor generation using the protocols described previously in this paper. Unfortunately, there are still instances where amalgam is drilled without some or all of these precautions. It would be anticipated that not using safety protocols would substantially increase the levels of mercury vapor generated from particulate and increase the risk of mercury absorption.

The average sized filling contains approximately 500 mg of mercury [44]. This filling would be comparable to a 2 rated filling in the size scoring that was adopted in this paper. The amount of particulate generated is, of course, determined by not only the size of the filling but also the technique used to drill the tooth. Using a smaller sized, sharp bur (drill bit) and removing the filling by cross hatching and removing as many solid chunks of amalgam as possible can reduce the amount of amalgam that is drilled upon.

Nevertheless, the mass of mercury in the particulate that was measured accounts for much less than 1% of the total mercury in the filling. Considering the median size score was 6, it can be estimated that the fillings removed in this case would be made up of approximately 1500 mg of mercury. The median mass of particulate is 1190 μg or 1.19 mg, which accounts for 0.08% of the total mercury in the fillings.

It is difficult to account for all of the particulate because the ultimate destination of it is varied. It is favorable for the majority of it to be taken up by the high-volume suction and collected in an amalgam separator. Other destinations include the lungs, skin, clothing/uniforms of the patient and the dental worker, surfaces of the operatory, the filters of an auxiliary evacuation, contamination of dental barriers, and contamination of dental instruments.

Answers to the research questions;

- Using all engineering controls to reduce Hg vapor, the highest concentration of Hg vapor measured in this study was 899 μg/m^3^.
- There were samples that continued to emit mercury vapor levels above PEL (100 μg/m^3^) for over 4 h. After 1 h, 17 of the 21 samples were still volatilizing Hg vapor that exceeded 10 μg/m^3^.
- Pearson coefficients and linear regressions were used to estimate correlations between three variables. Correlations between mass and peak Hg (positive), mass Hg and size rating (negative), and peak Hg and size rating (negative) were all found to be statistically insignificant.

## Conclusions

An unrealized, significant, localized mercury vapor source that may be present for hours after dental drilling on amalgam was identified in this study. We showed that micron amounts of amalgam particulate generated from dental high-speed drilling volatilized measurable amounts of mercury vapor that frequently breaches occupational safety thresholds. This occurred despite the fact that all feasible engineering controls were used to minimize mercury exposure. It can be reasonably assumed that not using the engineering controls used in this study will drastically increase this form of mercury exposure to both the dental worker and the dental patient.

Current standard occupational practices of room monitoring for mercury vapor and swiping a specific 10 cm × 10 cm area for contamination may understate the risk of mercury exposure to dental workers and dental patients when amalgam particulate is generated. The mercury exposure defined in this paper may explain why dental workers seem to incur mercury related health effects even when safety thresholds are not breached.

Although there was an association with the mass of the particulate collected and the peak levels of mercury vapor measured, it was not statistically significant, and we noted a fairly broad variability in the amount of Hg vapor emitted from a given mass of particulate. There are many functions in the operation of amalgam removal that can have the potential to affect the temperature of the particulate. Factors such as drill pressure, drill rpm, sharpness of the bur (bit), type of bit (diamond vs carbide), accuracy of the positioning of the suction, water temperature, accuracy of the water spray, water flow, the type of amalgam, the age of the filling, and the size of the particulate all may play a role in the variability of volatilization that we experienced.

## Recommendations

It is recommended that the engineering controls listed in this study be used at all times when amalgam is removed with a high-speed dental drill. In addition to these protocols, the authors have compiled a list of additional recommendations to reduce the risk of mercury exposure to dental workers and dental patients. These recommendations are available at Additional file 1.

## Further study

Further studies are required to determine the dispersal pattern of particulate during amalgam removal.

## Additional file

Additional file 1: Supplementary recommendations for dentists and dental schools to reduce mercury exposure from volatilizing amalgam particulate. (DOCX 20 kb)

## Data Availability

All data is available to the reviewers. The samples collected were destroyed in the analytical portion of the study.

## Abbreviations

AAS: Atomic Absorption Spectroscopy
ACGIH: Association Advancing Occupational and Environmental Health/American Conference of Governmental Industrial Hygienists
AIHS: Alberta Innovates – Health Solutions
AOHS: Alberta (Canada) Occupational Health and Safety
ARECCI: A Project Ethics Community Consensus Initiative
FDI: World Dental Federation
Hg: Mercury
kPa: Kilopascal
MSDS: Manufacturers Safety Data Sheet
NIOSH: The National Institute for Occupational Safety and Health
OSHA: Occupational Safety and Health Administration
PEL: Permissible Exposure Limit
REL: Recommended Exposure Limit
SDS: Safety Data Sheet
TLV: Threshold limit values
TWL: Time Weighted Level
WHO: World Health Organization
