# Supplementary material for: Mercury vapor volatilization from particulate generated from dental amalgam removal with a high-speed dental drill – a significant source of exposure
Source: J Occup Med Toxicol. 2019 Jul 17;14:22. doi: 10.1186/s12995-019-0240-2 (PMC6637613; doi:10.1186/s12995-019-0240-2)
Supplement: Supplementary file 1 — Supplementary recommendations for dentists and dental schools to reduce mercury exposure from volatilizing amalgam particulate. (DOCX 20 kb) [file 12995_2019_240_MOESM1_ESM.docx]

**SUPPLEMENTARY RECOMMENDATIONS FOR DENTISTS AND DENTAL SCHOOLS TO REDUCE MERCURY EXPOSURE FROM VOLATILIZING AMALGAM PARTICULATE**

The identification of the occupational mercury exposure from mercury vapor and particulate in this methodology has implications for dental practices and dental schools. Based on our findings, we recommend the following:

1) The risk of mercury exposure via volatilizing particulate when amalgam is drilled with a dental high-speed hand piece requires protection of inhalation points and mercury resistant coverage for skin of dental workers and dental patients.

2) Mercury volatilizing particulate generated from amalgam removal with a high-speed drill should be considered at least as dangerous as elemental mercury.

3) All engineering controls described in the methodology should be considered to reduce Hg vapor production and increase dental worker safety during amalgam removal. These include;

- copious amounts of water
- reduced drilling of the amalgam by cross hatching the material and removing bulk pieces
- high volume suction with custom isolation tip (Clean Up brand)
- secondary air evacuation
- non-latex dental dam on the patient
- full facial and body barrier for patient
- patient saliva suction behind the rubber dam
- alternative air supply to patient
- face shield, mercury rated gown and head protection, nitrile gloves, mercury rated breathing protection for dentist and assistant

4) Because there was no statistical difference identified in the size of the filling and the amount of vapor generated, any intervention that involves the drilling of amalgam fillings requires occupational protection.

5) Limit reusable supplies and tools that require sterilization.

6) Use commercial mercury binding agents on any surfaces, sundries or equipment that may capture amalgam particulate.

7) When possible, eliminate or reduce the amount of drilling on amalgam.

8) During removal and while contaminated suction tips are present, leave the suction on to evacuate the Hg vapor from the particulate collected on the tip.

9) Ensure that the high-volume suction unit is working adequately.

10) In cases where the amalgam containing tooth is being removed (extracted), consider removing only tooth structure so the amalgam filling can be retrieved in one piece and disposed of in a conscientious manner.

11) When the remaining tooth structure is significantly undermined on an amalgam-containing tooth, consider removing tooth structure that will give access to the amalgam, so it may be retrieved without drilling on it. This is quite frequent in teeth that have received or are receiving root canal therapy.

12) Discontinuing the placement of amalgam fillings, especially considering the availability of suitable alternatives, may be the most important step in reducing the societal load of mercury containing restorations. This will reduce the dental worker’s greatest source of mercury contamination. Discontinuing the placement of amalgam fillings will also be beneficial to the environment, as the waste from installation of amalgam fillings represents a significant load into the environment. The fact that amalgam contains mercury means that the product must be stored in a protective capsule and must be pre-dosed to reduce the handling of the product. The protective measure of the capsule, however, creates another route of pollution because these contaminated one-time use capsules require conscientious disposal, and many dental practices do not have a system in place for such proper disposal.

13) One can use a technique when replacing amalgam fillings where avoidance of drilling on amalgam is chosen. In this technique, the dentist can drill the surrounding tooth structure adjacent to the filling to allow retrieval of the amalgam filling. The drawback of this technique is that viable tooth structure has to be sacrificed in order to access the filling being replaced, which in turn reduces the structural integrity of the tooth.

14) If installation of amalgam filling material continues, then it would be prudent to include in the patient informed consent that the costs, the health risks of removal, and the potential loss of tooth structure have to be considered before installation.

15) The extended length of time that particulate discharges mercury vapor is pertinent for the management of contaminated barriers and sundries involved in processes that involve the drilling of amalgam. In order to minimize exposure into the room air, it would be advisable to remove any contaminated disposables from the building and allow them to off-gas into the outside atmosphere, reducing the risk of elevating room mercury air levels. This poses a bigger problem in that, while protecting dental workers by moving a dangerous substance out of the work place, dental practices doing so add to the burden of global air pollution. The practice of placing disposable mercury contaminated sundries and protective equipment in a re-openable vessel becomes ill-advised. This is because every time the lid is opened, there is a risk of the Hg vapor bolus to emanate from the vessel, which has the potential to be inhaled or spilled into the general room air, increasing its overall concentration.

16) Design monitoring systems for dental workers that fully and accurately measure the full risk of Hg exposure, including that generated from particulate during and after amalgam removal.
